# SOX2 suppresses *CDKN1A* to sustain growth of lung squamous cell carcinoma

**DOI:** 10.1038/srep20113

**Published:** 2016-02-05

**Authors:** Takuya Fukazawa, Minzhe Guo, Naomasa Ishida, Tomoki Yamatsuji, Munenori Takaoka, Etsuko Yokota, Minoru Haisa, Noriko Miyake, Tomoko Ikeda, Tatsuo Okui, Nagio Takigawa, Yutaka Maeda, Yoshio Naomoto

**Affiliations:** 1 Department of General Surgery,Okayama, Japan, 700-8505; 2 Department of General Internal Medicine 4,Okayama, Japan, 700-8505; 3 Kawasaki Hospital Research Unit, Kawasaki Medical School, Okayama, Japan, 700-8505; 4 Department of Electrical Engineering and Computing Systems, University of Cincinnati, Cincinnati, Ohio, 45221; 5 Division of Pulmonary Biology, Cincinnati Children’s Hospital Medical Center, Cincinnati, Ohio, 45229-3039; 6 Division of Hematology and Oncology, Indiana University School of Medicine, Indianapolis, Indiana, 46202.

## Abstract

Since the *SOX2* amplification was identified in lung squamous cell carcinoma (lung SCC), SOX2 transcriptional downstream targets have been actively investigated; however, such targets are often cell line specific. Here, in order to identify highly consensus SOX2 downstream genes in lung SCC cells, we used RNA-seq data from 178 lung SCC specimens (containing tumor and tumor-associated cells) and analyzed the correlation between *SOX2* and previously-reported *SOX2*-controlled genes in lung SCC. In addition, we used another RNA-seq dataset from 105 non-small cell lung cancer cell lines (NSCLC; including 4 lung SCC cell lines) and again analyzed the correlation between *SOX2* and the reported *SOX2*-controlled genes in the NSCLC cell lines (no tumor-associated cells). We combined the two analyses and identified genes commonly correlated with *SOX2* in both datasets. Among the 99 genes reported as SOX2 downstream and/or correlated genes, we found 4 negatively-correlated (e.g., *CDKN1A*) and 11 positively-correlated genes with *SOX2*. We used biological studies to demonstrate that *CDKN1A* was suppressed by SOX2 in lung SCC cells. G1 cell cycle arrest induced by *SOX2* siRNA was rescued by *CDKN1A* siRNA. These results indicate that the tumorigenic effect of SOX2 in lung SCC cells is mediated in part by suppression of *CDKN1A*.

Lung squamous cell carcinoma (lung SCC) is the second most frequent type of non-small cell lung carcinoma (NSCLC) after lung adenocarcinoma (lung AC)^1^. In contrast to the recent discovery of targeted therapies for lung AC, including *EGFR* mutant or *ALK* fusions, there is no effective therapy for lung SCC other than chemotherapy^2^,^3^,^4^. In order to understand the molecular pathogenesis that leads to identifying potential therapeutic molecular targets for lung SCC, extensive genetic analysis, including next-generation sequencing, have been performed, which has revealed amplification of *SOX2, PIK3CA, PDGFRA, BRF2* and *FGFR1*, deletion of *CDKN2A/B, PTEN, TP53* and *NF1*, and mutations of *PIK3CA, NRF2, DDR2, PTEN, EPHA2, LKB1* and *AKT1* in lung SCC^5^,^6^. The genomic amplification of *SOX2* is seen in 20% of lung SCC^7^ while increased expression of SOX2 is seen in 90% of lung SCC^8^, suggesting that SOX2 mediates a major tumorigenic effect on lung SCC regardless of genetic alterations.

SOX2 plays an oncogenic role not only in lung SCC but also in other cancers, including lung AC, ovarian, breast, esophageal, gastric, colon and pancreatic cancers^9^,^10^,^11^,^12^,^13^. SOX2 is a transcription factor, thus SOX2 downstream genes that exert a tumorigenic effect have been actively sought in such different types of cancers (summarized in Table 1). However, due to its relatively recent finding as an oncogene^11^, consensus SOX2 downstream targets that bear a tumorigenic function have not been established yet. In the present study, we utilized gene expression data from The Cancer Genome Atlas (TCGA) human lung SCC samples (n = 178)^14^ and determined a correlation in lung SCC between *SOX2* and previously-reported *SOX2* downstream targets in the multiple cancer cell lines. The limitation of using this TCGA lung SCC dataset is that the expression of each gene in the dataset is comprised of the combined gene expression profiles of tumor cells and tumor-associated endothelial cells, fibroblasts and immune cells, which hampers the identification of tumor cell-specific gene-to-gene correlations. Thus, we also utilized another gene expression dataset from non-small cell lung cancer (NSCLC) cell lines (n = 105), including 4 lung SCC cell lines^15^, and assessed the correlation between SOX2 and the reported SOX2 downstream targets in the NSCLC cell lines. The limitation of using this NSCLC cell line dataset is that it includes not only lung SCC cell lines but also other lung carcinoma cell lines (e.g., lung AD cell lines). Thus, after we analyzed the two datasets, we selected genes that were commonly correlated with *SOX2* in both datasets, which would likely be *SOX2*-correlated genes specific to lung SCC cells. Among 99 genes identified in previous reports as regulated by or correlated with SOX2 in multiple cancer cell lines, the expression of only 15 genes was positively or negatively correlated with the expression of *SOX2* in both the 178 lung SCC specimens and the 105 NSCLC cell lines. Among the 15 genes, CDKN1A (also known as p21[Cip1/Waf1]) that induces G1 cell cycle arrest was determined by RNA interference and adenovirus-mediated ectopic expression experiments to be a negative downstream target of SOX2 in multiple lung SCC cell lines. G1 cell cycle arrest induced by the reduction of SOX2 was reinstated by the reduction of CDKN1A in lung SCC cell lines, indicating that CDKN1A is an intrinsic SOX2 target influencing tumorigenicity in lung SCC cells. Here, we report that CDKN1A is a highly consensus gene target of the oncogenic transcription factor SOX2 in lung SCC cells.

## Results

In order to identify highly consensus SOX2 downstream genes in lung SCC cells, we investigated genes previously reported to be regulated by SOX2 in multiple cancer cell lines. As shown in the Table 1, SOX2 regulates cell cycle-related genes positively or negatively. CDKN1A, which induces G1 arrest, is repressed by SOX2 in A549 lung carcinoma cells, pancreatic cancer cells^13^,^16^ and gastric cancer cells^17^. CDKN1B, which also induces G1 arrest, is repressed by SOX2 in pancreatic cancer cells and gastric cancer cells. CCND1, which accelerates cell cycle, is activated by SOX2 in gastric cancer cells and MCF7 breast cancer cells^10^,^17^. Overall, SOX2 represses cell cycle inhibitors and activates cell cycle accelerators; however, the pattern of gene regulation is not universal in different cancer cell types. Additionally, genes comprising the WNT, NOTCH, RAS, TGF/BMP and EMT pathways that are involved in cancer progression and metastasis are regulated by SOX2 in multiple cancer cell lines^9^,^16^,^18^,^19^,^20^ (summarized in Table 1). In addition to genes identified as regulated by SOX2 in previous reports, we hypothesized that the previously reported top 50 genes positively correlated with SOX2 expression in stage I/II lung SCC^11^ might be directly regulated by SOX2 in lung SCC cells. In total, we selected 99 genes that might be commonly regulated by SOX2 in lung SCC cells (Table 1).

**Figure 1:**
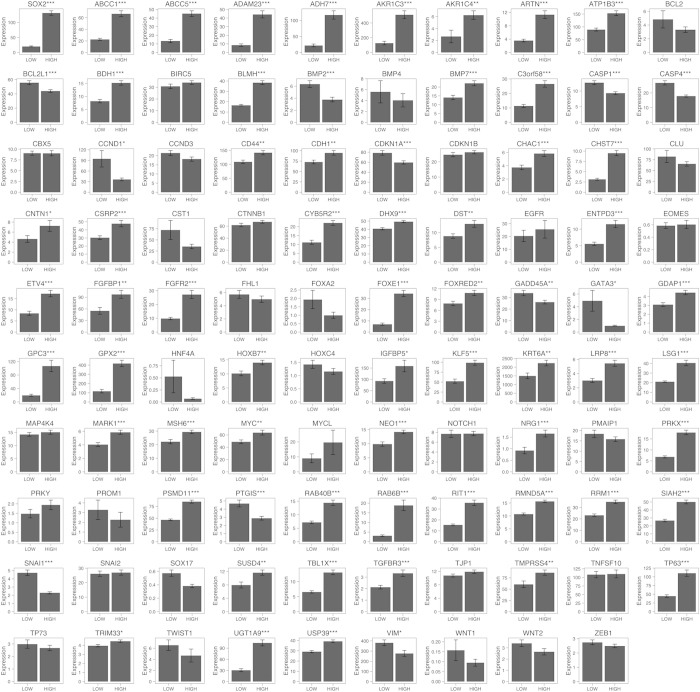
*SOX2* positively or negatively correlated genes in the human lung squamous cell carcinoma (SCC) samples from the TCGA. The Cancer Genome Atlas (TCGA) human lung squamous cell carcinoma (SCC) samples (n = 178) were divided into two groups that have high RNA expression of *SOX2* (HIGH, n = 89) or low expression of *SOX2* (LOW, n = 89). Two-tailed Welch’s t-test was used to determine the significance of gene expression between the two groups. Genes with significantly higher expression in the HIGH group (p < 0.05 and average expression > 1) were considered as *SOX2* positively correlated genes, while genes with significantly higher expression in the LOW group (p < 0.05 and average expression > 1) were considered as *SOX2* negatively correlated genes. The expression was measured in RPKM and quantile normalized. *PLA2G4B* was not present in the TCGA SCC dataset. Results represent the mean ± S.E.M. *indicates p < 0.05, **indicates p < 0.01, and ***indicates p < 0.001.

**Figure 2:**
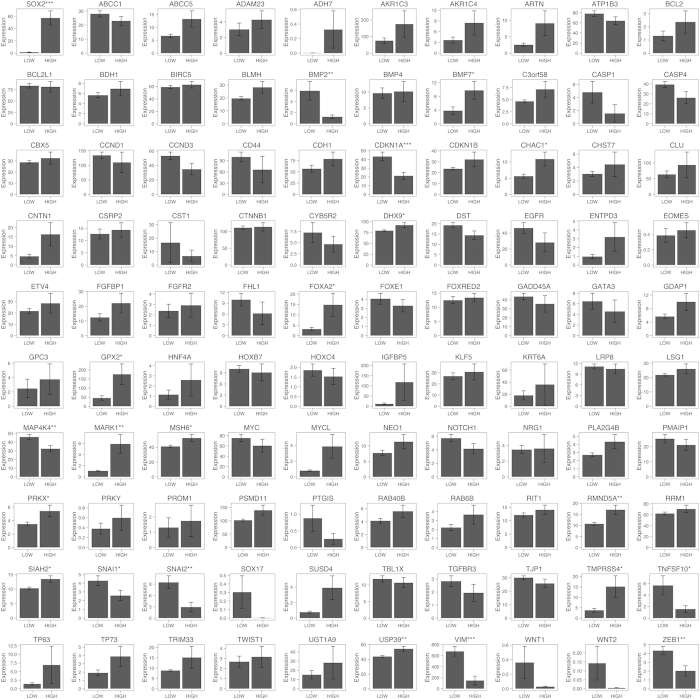
*SOX2* positively or negatively correlated genes in the human non-small cell lung cancer (NSCLC) cell lines. Human non-small cell lung cancer (NSCLC) cell lines (n = 105) were separated into two groups that have high RNA expression of *SOX2* (HIGH, n = 20) or low expression of *SOX2* (LOW, n = 85). The expression of previously reported SOX2 downstream or correlated genes was assessed as to whether the expression of those genes was correlated with that of SOX2. Two-tailed Welch’s t-test was used to determine the significance of gene expression between the two groups. Genes with significantly higher expression in the HIGH group (p < 0.05 and average expression >1) were considered as SOX2 positively correlated genes, while genes with significantly higher expression in the LOW group (p < 0.05 and average expression >1) were considered as SOX2 negatively correlated genes. The expression was measured in RPKM and quantile normalized. Results represent the mean ± S.E.M. *indicates p < 0.05, **indicates p < 0.01, and ***indicates p < 0.001.

Next, in order to determine which of the possible 99 genes are correlated with *SOX2* in lung SCC cells, we used a RNA-seq dataset from TCGA^14^ to determine whether the expression of any of the 99 genes is correlated with that of *SOX2* in 178 lung SCC specimens. The 178 lung SCC specimens were divided into two groups, *SOX2*-HIGH and *SOX2*-LOW groups, based on the median level of SOX2 expression in the lung SCC specimens (see Materials and Methods for details). Then, we assessed which genes are correlated with *SOX2*. As shown in Fig. 1, 57 genes (e.g., *BMP7*) were positively correlated with *SOX2* while 11 genes (e.g., *CDKN1A*) were negatively correlated with *SOX2* in lung SCC, suggesting that these genes might be regulated by SOX2 in lung SCC cells; however, it remains unknown whether the gene-to-gene correlation would be intrinsic or extrinsic since the TCGA lung SCC data contains gene expression from heterogeneous cell populations, including not only tumor cells but also tumor-associated cells. Thus, we used another RNA-seq dataset from 105 non-small cell lung cancer (NSCLC) cell lines (including 4 lung SCC cell lines)^15^ and performed the same analysis (see Materials and Methods for details). As shown in Fig. 2, 12 genes (e.g., *BMP7*) were positively correlated with *SOX2* while 8 genes (e.g., *CDKN1A*) were negatively correlated with *SOX2* in the NSCLC cells, suggesting that these genes might be regulated by SOX2 in lung SCC cells. Although the NSCLC cell lines do not contain tumor-associated cells, the gene-to-gene correlations might occur in non-lung SCC cells (e.g., lung AD cells). Thus, we combined the two analyses (Figs 1 and 2) and sought to identify most probable gene-to-gene correlation in lung SCC cells. Among the 99 genes, *BMP7, CHAC1, DHX9, GPX2, MARK1, MSH6, PRKX, RMND5A, SIAH2, TMPRSS4* and *USP39* were positively correlated while *BMP2, CDKN1A, SNAI1* and *VIM* were negatively correlated with *SOX2* in both datasets, suggesting that these genes are intrinsically regulated by SOX2 in lung SCC cells.

**Figure 3:**
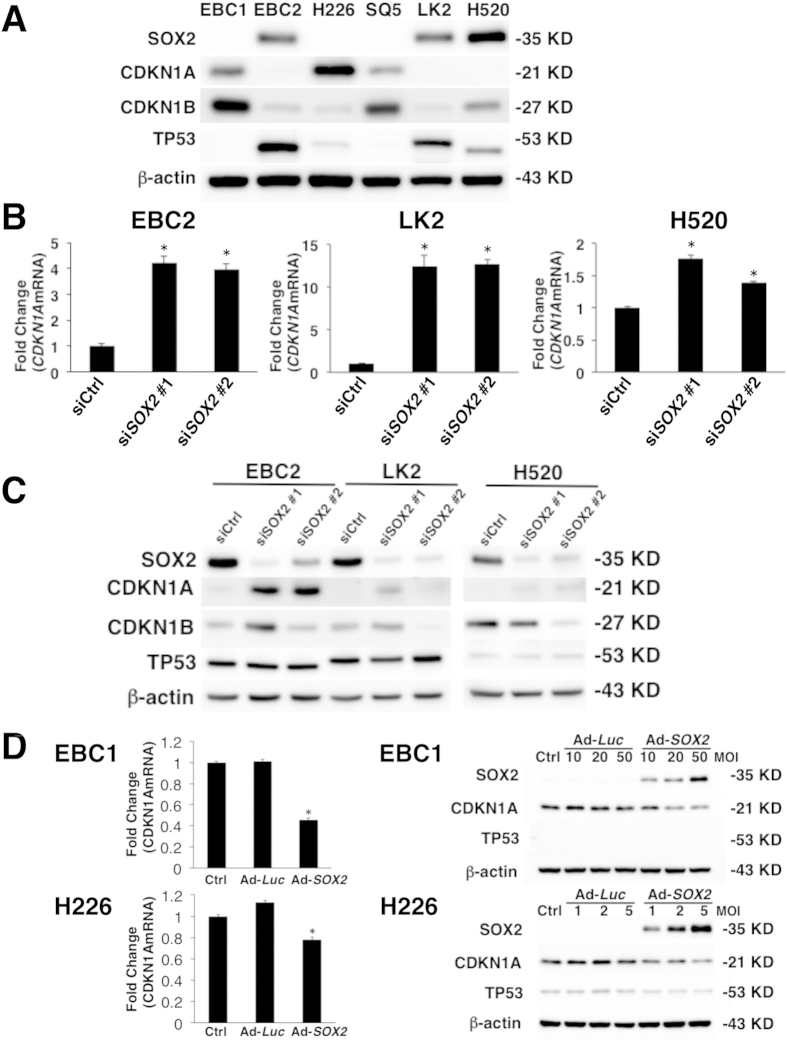
SOX2 suppresses CDKN1A in human lung SCC cells. (**A**) Immunoblot analysis of SOX2, CDKN1A, CDKN1B and TP53 in the indicated lung SCC cell lines. The expression level of β-actin is shown as a control. (**B**) qPCR demonstrated that *CDKN1A* mRNA was significantly increased after SOX2 silencing by *SOX2* siRNAs (si*SOX2* #1 and si*SOX2* #2) in EBC2, LK2 and H520 lung SCC cells. Non-targeting siRNA was used as a control (siCtrl). Detection of *GAPDH* was used for normalization. Results represent the mean ± SD (n = 3). Statistical significance was defined as p < 0.01 (*). (**C**) Immunoblot analysis shows increased CDKN1A expression 48** **hours after *SOX2* siRNAs (si*SOX2* #1 and si*SOX2* #2) transfection in EBC2, LK2 and H520 lung SCC cells. TP53 expression was not changed after *SOX2* siRNAs transfection. Non-targeting siRNA was used as control (siCtrl). (**D**) The qPCR analysis showed that *CDKN1A* mRNA expression was decreased 48** **hours after Ad-CMV/*SOX2* (Ad-*SOX2*) infection at a MOI of 100 in EBC1 and at a MOI of 10 in H226 lung SCC cells. Ad-CMV/*Luc* (Ad-*Luc*) did not change *CDKN1A* mRNA expression in both lung SCC cell lines. Non-treated cells were used as a control (Ctrl). Immunoblot analysis demonstrated that CDKN1A protein was suppressed 48** **hours after Ad-*SOX2* infection in EBC1 and H226 lung SCC cells. Ad-*Luc* did not change CDKN1A protein expression in the cells. TP53 expression was not changed in the cells. Statistical significance was defined as p < 0.01 (*).

Next, in order to confirm whether the RNA-seq data analyses indeed reflect their correlation at the protein level in lung SCC cell lines, we performed western blotting using cell extracts from six lung SCC cell lines and determined the protein expression of SOX2 and two G1 cell cycle inhibitors CDKN1A and CDKN1B. We focused on these cell cycle genes since SOX2 has been shown by several groups to influence cell proliferation *in vitro*^13^,^16^. *CDKN1B* was repressed by SOX2 in pancreatic cancer cell lines^13^; however, a negative correlation with SOX2 was not observed in our RNA-seq data analyses (Figs 1 and 2). Consistent with our RNA-seq data analyses (Figs 1 and 2), lung SCC cell lines (EBC2, LK2 and H520) that had a high expression of SOX2 had no expression of CDKN1A at the protein level. Conversely, lung SCC cell lines (EBC1, H226 and SQ5) that had a low expression of SOX2 did express CDKN1A (Fig. 3A), indicating that the expression of SOX2 and CDKN1A is inversely related. In addition to the expression in the cell lines, we also investigated the expression of SOX2 and CDKN1A in human primary lung SCC specimens. As shown in Supplemental Fig. 1, the majority of lung SCC specimens expressing SOX2 did not express CDKN1A. As indicated in the RNA-seq data analysis (Figs 1 and 2), any correlation between SOX2 and CDKN1B in the various lung SCC cell lines was not observed at the protein level (Fig. 3A,C), which suggests that SOX2 regulates CDKN1B in a cell-type specific fashion (pancreatic cancer vs. lung SCC). A similar negative correlation between SOX2 and VIM that was indicated by the RNA-seq data analyses (Figs 1 and 2) was also observed at the protein level in lung SCC cell lines (Supplemental Figure 2A).

Next, in order to determine whether SOX2 indeed functionally regulates the expression of CDKN1A, we employed a RNA interference approach using *SOX2* siRNA. Knockdown of SOX2 by RNA interference has been shown to inhibit cell viability and colony formation of lung SCC cells, again indicating that SOX2 sustains cell proliferation^11^. We were able to reproduce the effect of SOX2 using two independent siRNAs in multiple lung SCC cell lines *in vitro* and *in vivo* (Supplemental Figure 3A, 3B and 3C). Then, we investigated using these siRNAs targeting SOX2 to determine whether suppression of SOX2 influences the expression of CDKN1A in lung SCC cells. As shown in Fig. 3B,C, CDKN1A but not CDKN1B was induced by the two independent siRNAs targeting SOX2 in multiple lung SCC cell lines at the mRNA and protein levels, indicating that SOX2 functionally suppresses the expression of *CDKN1A*. TP53 is known to induce *CDKN1A*^21^; however, the expression of TP53 was not influenced by *SOX2* siRNAs (Fig. 3C), indicating that SOX2 suppresses *CDKN1A* in lung SCC cells not through TP53. Additionally, *CDKN1A* was induced by SOX2 siRNAs in the presence of cycloheximide (Supplemental Fig. 4), suggesting the direct suppression of *CDKN1A* by SOX2, as has been reported in pancreatic cancer cells^13^. VIM and ZEB1 expression were also induced by these *SOX2* siRNAs in lung SCC cell lines (Supplemental Figure 2B, 2C), indicating that SOX2 functionally suppresses the expression of VIM and ZEB1. The negative correlation between *SOX2* and *ZEB1* was indicated by the bioinformatical analysis using the 105 NSCLC cell lines dataset but not the 178 lung SCC specimens dataset (Figs 1 and 2); however the biological analysis indicated that SOX2 intrinsically regulates *ZEB1* in lung SCC cells, suggesting the requirement of validating bioinformatical analysis by biological experiments. We further confirmed the effect of SOX2 using adenovirus-expressing SOX2. Adenovirus-mediated expression of SOX2 significantly suppressed the expression of CDKN1A at the mRNA and protein levels (Fig. 3D). These loss-of-function and gain-of-function experiments indicate that SOX2 negatively regulates the expression of the G1 cell cycle inhibitor CDKN1A in lung SCC cells.

**Figure 4:**
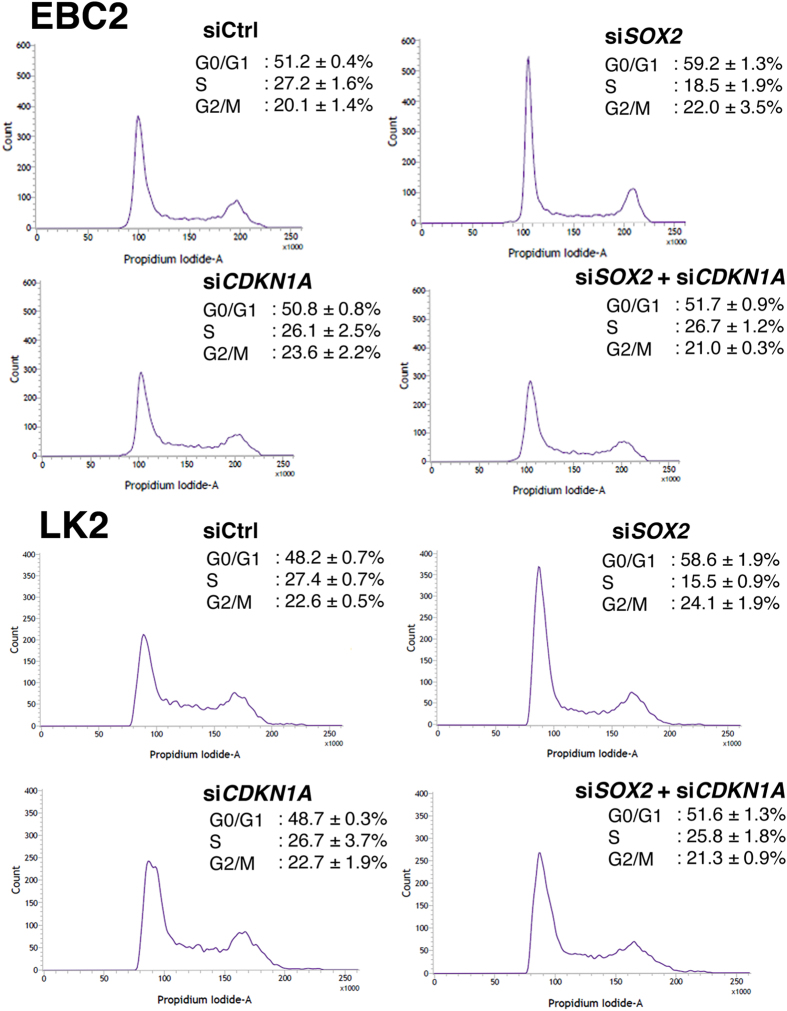
CD*KN1A* siRNAs released G1 cell cycle arrest induced by *SOX2* siRNA. Flow cytometry cell cycle analysis of EBC2 and LK2 lung SCC cells transiently transfected with non-targeting siRNA (siCtrl), *SOX2* siRNA (s*iSOX2*), *CDKN1A* siRNA (si*CDKN1A*) or the mixture of *SOX2* and *CDKN1A* siRNAs (si*SOX2* + si*CDKN1A*). Cells were harvested at 72** **hours after siRNA transfection. Indicated in the inset of each panel are the percentages of each phase of cell cycle, where values represent the mean ± SD of triplicate measurements.

In the next experiment, we sought to determine whether the increased expression of CDKN1A by *SOX2* siRNA alters the cell cycle. As shown in Fig. 4, *SOX2* siRNA induced the G1 cell cycle arrest in lung SCC (EBC2 and LK2) cells, presumably through the induced expression of CDKN1A. Inhibition of CDKN1A by siRNAs did not influence the cell cycle in EBC2 and LK2 cells (Fig. 4) presumably because they do not express endogenous CDKN1A (Fig. 2A). siRNAs targeting *CDKN1A* were validated using cells expressing endogenous CDKN1A (Supplemental Figure 5). We then investigated whether the G1 arrest induced by the *SOX2* siRNA is mediated by the increased expression of CDKN1A using the siRNAs targeting *CDKN1A*. When both *SOX2* siRNA and *CDKN1A* siRNA were transfected in EBC2 and LK2 cells, the G1 arrest induced by *SOX2* siRNA was restored to base level by the presence of the *CDKN1A* siRNA (Fig. 4), indicating that SOX2 sustains cell cycle by inhibiting the expression of CDKN1A, a G1 cell cycle inhibitor.

## Discussion

SOX2 has been recognized as a lineage-specific oncogenic transcription factor in lung SCC cells; however, consensus downstream genes of SOX2 have not been established yet. In the present study, we bioinformatically identified highly consensus downstream genes regulated by SOX2 in lung SCC cells by combining previously-reported SOX2 downstream/correlated genes in multiple cancer cell lines with two RNA-seq datasets from 178 lung SCC specimens and 105 NSCLC cell lines. We further biologically validated *CDKN1A, VIM* and *ZEB1* as intrinsic SOX2 downstream genes in lung SCC cells by loss-of-function and gain-of-function biological experiments.

Unlike SOX2, downstream gene targets of NKX2-1 (also known as TTF-1), another lineage-specific oncogenic transcription factor in lung AC, are well known. Due to a lung defect in *Nkx2-1* knockout mice^22^, NKX2-1 has been implicated in the regulation of lung specific genes, including surfactant protein genes^23^. Recent *in vitro* and *in vivo* studies using heterozygous or conditionally deleted *Nkx2-1* mice also indicate that NKX2-1 directly suppresses the expression of mucous genes in asthma and lung cancer^24^,^25^,^26^,^27^. Since *Sox2* knockout mice die in early embryonic development^28^, the role of SOX2 in lung had not been understood until recently. Conditional deletion of *SOX2* in lung epithelium in mice recently revealed that SOX2 is required for proliferation/differentiation of airway epithelial cells^29^,^30^. In addition to the mouse data, the amplification of *SOX2* in human lung SCC^31^,^32^ clearly indicates that SOX2 regulates genes involved in normal lung morphogenesis and lung SCC. Since SOX2 controls tumorigenesis in multiple cancers in addition to lung SCC, multiple groups have reported SOX2 downstream genes that are involved in tumor growth in different cancer types. However, such analyses are often based on a limited number of cancer cell lines, thus the proposed SOX2 targets are not regulated by SOX2 in other cell lines that are not included in the analyses. In order to obtain highly conserved SOX2 downstream targets instead, we decided to investigate gene expression profiles of a large number of lung SCC specimens combined with NSCLC cell lines, which would identify genes positively/negatively correlated with SOX2 universally in lung SCC cells. The RNA-seq data of the 105 NSCLC cell lines, including 4 lung SCC cell lines, were obtained from 675 human cancer cell lines, including non-NSCLC cell lines, reported by Klijn *et al.*^15^. Ideally, gene expression profiles of only lung SCC cell lines should be employed; however, due to the limited number of definitively classified lung SCC cell lines (4 cell lines), we include the entire 105 NSCLC cell lines, including not definitively classified cell lines such as “lung carcinoma” and “non-small cell lung carcinoma”, for the analysis. However, in the 4 lung SCC cell lines, the mean expression of *CDKN1A* (expression = 27.610) in *SOX2*-LOW group (LOU-NH91 and SK-MES-1) was higher than the mean expression of *CDKN1A* (expression = 5.787) in the *SOX2*-HIGH group (NCI-H2170 and NCI-H520), indicating that *SOX2* is inversely related with *CDKN1A* even in this limited number of lung SCC cell lines. The results were further biologically validated using multiple lung SCC cell lines, suggesting that our approach using gene expression profiles of the 105 NSCLC cell lines provide statistical power to identify potential genes correlated with *SOX2*. In addition to determining SOX2 downstream genes in lung SCC cell, our approach will be useful for identifying genes regulated by SOX2 in other cancer cells or genes regulated by any other transcription factors in multiple cancer cells.

In addition to *CDKN1A, BMP2, SNAI1* and *VIM* were also negatively correlated with *SOX2* in our data analysis. *VIM* and *SNAI1* are known to induce Epithelial-Mesenchymal Transition (EMT). The EMT is understood as a process that induces tumor invasion and metastasis^33^,^34^. The loss-of-function study showed that SOX2 inhibited VIM and another EMT-related factor ZEB1 *in vitro*. These results suggest a dual-role of SOX2, in which SOX2 suppresses lung SCC invasion and metastasis (tumor suppressor) while SOX2 also promotes lung SCC proliferation (oncogene). Further investigation *in vitro* and *in vivo* is required to elucidate this dual function of SOX2 in lung SCC tumorigenesis. TNFSF10 (also known as TRAIL) that was negatively correlated with *SOX2* in the 105 NSCLC cell lines (Fig. 2) but not in the 178 lung SCC specimens is known to induce apoptosis; however, *SOX2* siRNA did not induce apoptosis in the lung SCC cell lines that we investigated (Supplemental Figure 6), suggesting that TNFSF10 may be a SOX2 downstream gene in non-lung SCC cells. These results indicate that combining multiple RNA-seq datasets with biological experiments is useful to identify unbiased transcriptional downstream targets and their associated biological functions.

Among the genes whose expression that we identified as positively correlated with SOX2 (*BMP7, CHAC1, DHX9, GPX2, MARK1, MSH6, PRKX, RMND5A, SIAH2, TMPRSS4* and *USP39*) in our bioinformatical analysis using both the 178 lung SCC specimens and 105 NSCLC cell lines, all of them were originally reported as top 50 genes that are positively correlated with *SOX2* in stage I/II lung SCC^11^, indicating that our approach is valid and useful to identify gene correlation and potential downstream targets in lung SCC cells. Further biological analysis is required to determine whether the above 11 genes are functionally relevant to SOX2 in lung SCC cells.

Our and others’ data clearly indicate that inhibition of SOX2 suppresses lung SCC growth *in vitro* and *in vivo*. However, our present data indicate that the suppression of lung SCC growth by SOX2 inhibition is mediated by cell cycle arrest (via CDKN1A induction) but not apoptotic cell death, suggesting that inhibition of SOX2 may not eliminate lung SCC. In addition, SOX2 inhibition slows lung SCC growth and may therefore lead to tumor resistance to chemotherapy that targets rapidly dividing cells. Furthermore, SOX2 inhibition induced genes related to EMT, which may cause tumor invasion and metastasis. Thus, targeting of SOX2 as a therapy for lung SCC should be taken cautiously. So far, SOX2 has been reported as a better prognosis marker for lung SCC by three groups and a worse prognosis marker by one group^8^,^35^,^36^,^37^,^38^. Identification of therapeutic targets to induce cell death and/or inhibit EMT in addition to targeting SOX2 may be required to eradicate SOX2-expressing lung SCC.

In summary, we bioinformatically combined previous reports on SOX2 gene regulation in multiple cancer cells with two gene expression datasets from 178 lung SCC specimens and 105 NSCLC cell lines and identified SOX2 downstream genes, one of which influences cell growth of multiple lung SCC cells. Further bioinformatical approaches, non-biased by previous reports, will make it possible to identify additional SOX2 downstream targets, which will lead to full understanding of the mechanisms of growth of lung SCC that harbors *SOX2* amplification and/or highly expressed SOX2 and identify potential therapeutic molecular targets to eradicate lung SCC.

## Materials and Methods

### Bioinformatical determination of *SOX2* positively or negatively correlated genes in the TCGA human lung squamous cell carcinoma (SCC) samples

The RNA-seq data of human lung squamous cell carcinoma (n = 178) were downloaded from The Cancer Genome Atlas (TCGA) data portal https://tcga-data.nci.nih.gov/docs/publications/lusc_2012/gaf.gene.rpkm.20111213.csv.zip. Expression values were measured in RPKM. For details on the original processing of the data, refer to the Supplemental Information of the original paper^14^. Expression values were quantile normalized using the “normalize.quantiles” function in the Bioconductor package preprocessCore (http://www.bioconductor.org/packages/release/bioc/html/preprocessCore.html). We divided the 178 samples into two groups based on their *SOX2* expression values; the *SOX2-*HIGH group contained 89 lung SCC samples with their *SOX2* expression greater than the median in the 178 samples (expression >55.594); and the remaining 89 lung SCC samples comprised the *SOX2*-LOW group. Two-tailed Welch’s t-test was used to determine the significance of gene expression between the two groups. Genes with significantly higher expression in *SOX2*-HIGH group (p < 0.05 and average expression >1) were considered as *SOX2* positively correlated genes, while genes with significantly higher expression in *SOX2*-LOW group (p < 0.05 and average expression >1) were considered as *SOX2* negatively correlated genes.

### Bioinformatical determination of *SOX2* positively or negatively correlated genes in the human non-small cell lung cancer (NSCLC) cell lines

The RNA-seq data of human cancer cell lines (n = 675) were downloaded from ArrayExpress using accession code E-MTAB-2706 (https://www.ebi.ac.uk/arrayexpress/experiments/E-MTAB-2706). 105 out of 675 samples were selected as non-small cell lung cancer (NSCLC) cell lines for analysis based on the following sample annotations; “OrganismPart” is lung and “Disease” is lung carcinoma, lung adenocarcinoma, lung anaplastic carcinoma, non-small cell lung carcinoma, squamous cell lung carcinoma, large cell lung carcinoma, lung mucoepidermoid carcinoma, lung papillary adenocarcinoma, lung adenosquamous carcinoma, bronchoalveolar adenocarcinoma, or squamous cell carcinoma. Among the 105 NSCLC cell lines, four were lung SCC cell line, including SK-MES-1, NCI-H520, NCI-H2170 and LOU-NH91. Expression values were measured in RPKM. For details on the original processing of the data, refer to the original paper^15^. The expression values were quantile normalized using the “normalize.quantiles” function in the Bioconductor package preprocessCore. We divided the 105 NSCLC cell lines into two groups based on their *SOX2* expression; *SOX2*-HIGH group contained 20 NSCLC cell lines with their *SOX2* expression greater than the mean in the 105 NSCLC cell lines (expression >12.195; e.g., NCI-H2170 and NCI-H520 lung SCC cell lines); and the remaining 85 NSCLC cell lines comprised *SOX2*-LOW group (e.g., LOU-NH91 and SK-MES-1 lung SCC cell lines). Two-tailed Welch’s t-test was used to determine the significance of gene expression between the two groups. Genes with significantly higher expression in *SOX2*-HIGH group (p < 0.05 and average expression >1) were considered as *SOX2* positively correlated genes, while genes with significantly higher expression in *SOX2*-LOW group (p < 0.05 and average expression >1) were considered as *SOX2* negatively correlated genes.

### Cell Lines and Culture Conditions

The human lung SCC cells H520, H226, the human lung carcinoma cells A549 and human foreskin fibroblast HFF1 were obtained from the American Type Culture Collection (Manassas, VA) and grown in RPMI 1640 (H520 and H226) or high glucose Dulbecco’s modified Eagle medium (A549 and HFF1) supplemented with 10% heat-inactivated fetal bovine serum. The lung SCC cells EBC1, EBC2, SQ5, LK2 were kindly provided by Dr. Kiura Katsuyuki (Department of Respiratory medicine, Okayama University Graduate School of Medicine and Dentistry, Okayama, Japan) and grown in RPMI 1640 supplemented with 10% heat-inactivated fetal bovine serum. All cell lines were cultured in 10% CO_2_ at 37 °C.

### Immunohistochemistry

Sections were sequentially deparaffinized through a series of xylene, graded ethanol, and water immersion steps. After being autoclaved in target retrieval solution (Dako, Carpinteria, CA, USA) for 15 minutes, sections were incubated with 3% hydrogen peroxide for 5 minutes to block endogenous peroxidase activity. A primary antibody specific for human SOX2 and CDKN1A was obtained from Cell Signaling Technology (Beverly, MA). Specimens were incubated overnight at 4 °C with a 1:50 dilution of SOX2 or CDKN1A antibody followed by three washes with TBS. The slides were treated with streptavidin-biotin complex (Envision System labeled polymer, horseradish peroxidase [HRP], Dako, Carpinteria, CA) for 60 minutes at room temperature. Immunoreactions were visualized using a 3,3′-diaminobenzidine (DAB) substrate-chromogen solution (Dako Cytomation Liquid DAB Substrate Chromogen System, Dako) and counterstained with hematoxylin. Sections were immersed in an ethanol and xylene bath and mounted for examination. For immunohistochemistry analysis, 40 lung SCC tissue sections were obtained from patients diagnosed with lung SCC who underwent surgical resection at Kawasaki Hospital, Okayama, Japan. The experimental protocol was approved by the Ethics Review Committee of Kawasaki Medical School (Ethics Committee reference number: 1310) and all experiments were performed in accordance with relevant guidelines and regulations of Kawasaki Medical School. The informed consent was obtained from all subjects.

### siRNA mediated inhibition of *SOX2* and *CDKN1A*

Cells were plated in a 6-well plate at a density of 3 × 10^5^ per well and cultured overnight at 37 °C. The following day 100 pmol of the different *SOX2* siRNA (#1: D-011778-01, #2: D-011778-02), *CDKN1A* siRNA (#1: D-003471-01, #2: 003471-02) or nontargeting siRNA (siCtrl; Thermo Scientific) were transfected using 7.5 μl of Lipofectamine RNAi MAX reagent (Invitrogen, Life Technologies, Carlsbad, CA) according to the manufacturer’s instructions. Incubation time for transfection reagents was 24 hours, at which time the medium was replaced with fresh regular medium. Cells were harvested 48 hours after transfection for immunoblotting or colony formation assays.

### Adenoviral vectors

The recombinant adenoviral vector Ad-CMV/SOX2 (Ad-*SOX2*) was obtained from VECTOR BioLabs (Malvern, PA) and the optimal multiplicity of infection (MOI) was determined by infecting each cell line with Ad-CMV/GFP and assessing the expression of GFP. The experimental protocol was approved by the institutional biosafety committee of Kawasaki Medical School (Reference number: 14-07) and carried out in accordance with the approved guidelines.

### Cell viability assay

Cells were plated in 12-well plates at a density of 1 × 10^5^ cells and cultured overnight at 37 °C. The following day 100 pmol of two different *SOX2* siRNA described above was transfected using 3.25 μl of Lipofectamine RNAi MAX reagent (Invitrogen, Life Technologies) according to the manufacturer’s instructions. Cells were harvested 48 hours after transfection and viable cells were assessed by a TC20 automated cell counter (Bio-Rad, Hercules, CA)^39^.

### Colony formation assay

Cells were plated in a 6-well plate at a density of 2 to 3 × 10^5^ per well and cultured overnight at 37 °C. The following day 100 pmol of different kinds of SOX2 siRNA described above were transfected using 7.5 μl of Lipofectamine RNAi Max reagent (Invitrogen, Life Technologies, Carlsbad, CA) according to the manufacturer’s instructions. Incubation time for transfection reagents was 24 hours, at which time the medium was replaced with fresh regular medium. Cells were harvested 48 hours after transfection, counted, plated in triplicate at a density of 5 × 10^2^ cells in 6-well plates for 14 days. The cells were then stained with Diff-Quik (Sysmex, Kobe, Japan)^40^. Colonies (a group of aggregated cells numbering at least 50) were then counted. The mean number of the control group was arbitrarily set to 100%, and all other numbers were normalized and percentage-specific cytotoxicity compared to colony formation in the control group was calculated.

### Immunoblot analysis

Cells were lysed in ice-cold M-PER lysis buffer purchased from Thermo Fisher Scientific (Rockford, IL). Cell lysates were clarified by centrifugation (20 min at 15,000 × g at 4 ^°^C) and protein concentration determined using the BCA protein assay (Thermo Fisher Scientific). Equal amounts of protein were separated on an SDS-PAGE gel. The gel was electrophoretically transferred to a Hybond PVDF transfer membrane (GE. Healthcare Ltd., Piscataway, NJ) and incubated with primary and secondary antibodies according to the Supersignal^®^ West Pico chemiluminescence protocol (Pierce, Rockford, IL). Antibody specific for β-actin was obtained from Sigma (St. Louis, MO) and antibody specific for SOX2, CDKN1A, CDKN1B, CDH1 and VIM were obtained from Cell Signaling Technology (Beverly, MA). Antibody specific for ZEB1 and TP53 were obtained from Santa Cruz Biotechnology (Santa Cruz, CA). Secondary horseradish peroxidase-conjugated antibodies were obtained from Jackson Immunoresearch Laboratories (West Grove, PA).

### Real time PCR

Total RNA from the cultured cells was obtained using TRIzol (Invitrogen, Life Technologies, Carlsbad, CA). Two μg of total RNA was used for reverse transcription. Reverse transcription was performed at 37 °C for 15 min using PrimeScript RT reagent Kit (Takara Bio, Inc., Shiga, Japan). Specific probes for *SOX2* (Hs01053049_s1), *CDKN1A* (Hs00355782_m1) and Glyceraldehyde-3-phosphate dehydrogenase (*GAPDH*) (Hs03929097_g1) for TaqMan Gene Expression Assays were obtained from Applied Biosystems (Life Technologies, CA). The real-time PCR reactions were carried out in a 48-well microtiter plate using the TaqMan Gene Expression Master Mix (Applied Biosystems). The reaction was performed in triplicate for each sample. The fluorescence of the PCR products was detected by TaqMan One-Step RT-PCR. The number of cycles for the amplification plot to reach the threshold limit (Ct value) was used for quantification. *GAPDH* was used as endogenous control.

### Flow cytometric analysis for cell cycle and apoptosis

For cell cycle analysis, cells were plated in 6-well plates at a density of 2 × 10^5^ cells per well and cultured overnight at 37 °C. The following day 100** **pmol of *SOX2* siRNA #1 and/or *CDKN1A* siRNA #1 was transfected using 7.5 μl of Lipofectamine RNAi Max reagent (Invitrogen, Life Technologies, Carlsbad, CA) according to the manufacturer’s instructions. Nontargeting siRNA (siCtrl) was used as a control. 48** **hours after transfection, cells were harvested and washed once with PBS. Cells were resuspended in PBS containing 0.2% Triton X-100 and 1** **mg/ml RNase for 5** **min at room temperature and then stained with propidium iodide at 50** **μg/ml to determine DNA cell cycle using a FACS Verse (BD Bioscience, San Jose, CA). Doublets, cell debris, and fixation artifacts were gated out, and DNA cell cycle was determined using FACSuite Version 1.0.2.2238. To detect apoptosis, 100** **pmol of *SOX2* siRNA #1 or #2 was transfected. Cells were analyzed by flow cytometry 48** **hours after transfection staining with AnnexinV-FITC conjugate (BD Bioscience) and propidium iodide according to manufacturer’s protocol. Non-targeting siRNA (siCtrl) was used as control.

### Mouse experiments

LK2 or EBC2 lung squamous carcinoma cells were plated in 10 cm dishes at a density of 4 × 10^6^ per dish and cultured overnight at 37 °C. The following day 580 pmol of the two different *SOX2* siRNA described above or nontargeting siRNA (siCtrl; Thermo Scientific) was transfected using 43 μl of Lipofectamine RNAi Max reagent (Invitrogen, Life Technologies, Carlsbad, CA) according to the manufacturer’s instructions. Incubation time for transfection reagents was 24 hours, at which time medium was replaced with fresh regular medium. Cells were harvested and washed once with PBS and then resuspended in culture medium. Human lung cancer xenografts were established in 6-wk-old female BALB/c nude mice (Charles River Laboratories Japan, Kanagawa, Japan) by subcutaneous (s.c.) inoculation of siRNA (*SOX2* #1, *SOX2* #2 or non-targeting) transfected LK2 or EBC2 lung SCC cells (2 × 10^6^ cells/200 μl) into the right dorsal flank. Tumors were measured two times a week, and tumor volume was calculated as a x b^2^ × 0.5, where a and b were large and small diameters, respectively. The experimental protocol was approved by the Ethics Review Committee for Animal Experimentation of Kawasaki Medical School (Ethics Committee reference number: 14-005) and carried out in accordance with the approved guidelines.

### Statistical analysis

Statistically significant differences between means and medians of the study groups were evaluated using Student’s t-test except for Figs 1 and 2. Statistical significance was defined as p < 0.01 (*).

## Additional Information

**How to cite this article**: Fukazawa, T. *et al.* SOX2 suppresses *CDKN1A* to sustain growth of lung squamous cell carcinoma. *Sci. Rep.* **6**, 20113; doi: 10.1038/srep20113 (2016).

## Supplementary Material

Supplementary Information

## Figures and Tables

**Table 1:** Previously reported SOX2 downstream genes in different cancer cell.

	Lung squamous cellcarcinoma cell lines(Fukazawa ***et al*****., this study)**	Pulmonary adeno carcinomaH2009 cells(Bass ***et al*****., 2014. Ref:** 11)	Lung squamous cell carcinoma LK2 and H520 cells (Fang ***et al*****., 2014. Ref:** 18)	Lung carcinoma A549 and CL1-0 cells (Chen ***et al*****.,2012, 2014. Ref:** 15, 17)	Lung carcinoma A549 and CL1-0 cells (Chou ***et al*****., 2013. Ref:** 41)	Lung squamouscell carcinoma (Hussenet ***et al*****., 2010. Ref:** 7)	Lung, ovarian and breast cancer cell lines(Zhang ***et al*****., 2014. Ref:** 9)	Breast cancer MCF7 cells (Chen ***et al*****., 2008. Ref:** 10)	Esophageal cancer KYSE70 cells (Watanabe ***et al*****., 2014. Ref:** 42)	Gastric cancer cell lines (Otsubo ***et al*****., 2013. Ref:** 16)	Colon cancer SW620 cells (Han ***et al*****., 2012. Ref:** 19)	Pancreatic cancer cell lines (Herreros-Villanueva ***et al*****., 2013. Ref:** 13)	Melanoma cell lines (Santini ***et al*****., 2014. Ref:** 43)
CDKN1A (p21)	repress			repress						repress		repress	
CDKN1B (p27)	No change									activate		repress	
CCND1								activate		repress			
CCND3												activate	
CTTNB1				activate							activate		
MYC				activate									
WNT1				activate									
WNT2				activate									
NOTCH1				activate									
MAP4K4				repress									
PMAIP1				activate									repress
TP73													repress
GADD45A													repress
BCl2													activate
BCL2L1					activate								
BIRC5													
TP63		activate											
KRT6A		activate											
EGFR					activate								
ETV4									activate				
FOXA2							repress						
HNF4A							repress						
EOMES							repress						
SOX17							repress						
BMP2							repress						
BMP4			repress										
CDH1	No change										repress	repress	
TJP1												repress	
VIM	repress										activate		
ZEB1	repress										activate		
SNAI1											activate		
SNAI2											activate		
TWIST1												activate	
PROM1												activate	
CD44												activate	
ALDH1												activate	
CBX5						activate							
GATA3						activate							
MYCL						activate							
HOXB7						activate							
HOXC4/HOXC6						activate							
RIT1						activate							
IGFBP5						activate							
NRG1						activate							
FGFBP1						activate							
HPLH1						repress							
PTGIS						repress							
CASP1						repress							
CASP4						repress							
CLU						repress							
TGFBR3						repress							
TNFSF10						repress							
