# Supplementary material for: SOX2 suppresses CDKN1A to sustain growth of lung squamous cell carcinoma
Source: Sci Rep. 2016 Feb 5;6:20113. doi: 10.1038/srep20113 (PMC4742851; doi:10.1038/srep20113)
Supplement: Supplementary Information [file srep20113-s1.pdf]

Supplementary Figures for

SOX2 suppresses *CDKN1A* to sustain growth of lung squamous cell carcinoma

Takuya Fukazawa[1], Minzhe Guo[4, 5], Naomasa Ishida[1], Tomoki Yamatsuji[1], Munenori Takaoka[1], Etsuko Yokota[1], Minoru Haisa[1], Noriko Miyake[3], Tomoko Ikeda[3], Tatsuo Okui[6], Nagio Takigawa[2], Yutaka Maeda[5] and Yoshio Naomoto[1]

[1]Department of General Surgery, [2]Department of General Internal Medicine 4, [3]Kawasaki Hospital Research Unit, Kawasaki Medical School, Okayama, Japan, 700-8505; [4]Department of Electrical Engineering and Computing Systems, University of Cincinnati, Cincinnati, Ohio, 45221; [5]Division of Pulmonary Biology, Cincinnati Children's Hospital Medical Center, Cincinnati, Ohio, 45229-3039; [6]Division of Hematology and Oncology, Indiana University School of Medicine, Indianapolis, Indiana, 46202

Co-corresponding authors:
Takuya Fukazawa, M.D., Ph.D.
Department of General Surgery, Kawasaki Medical School
2-1-80 Nakasange, Okayama 700-8505, Japan
Phone: +81-86-225-2111; Fax: +81-86-232-8343
E-mail: Fukazawat@aol.com

Yutaka Maeda, D.V.M, Ph.D.
Perinatal Institute, Division of Neonatology, Perinatal and Pulmonary Biology
Cincinnati Children's Hospital Medical Center and the University of Cincinnati College of Medicine
Cincinnati, Ohio 45229, USA
Tel: +1-513-636-3322; Fax: +1-513-636-7868
E-mail: yutaka.maeda@cchmc.org

Supplemental Figure 1

A

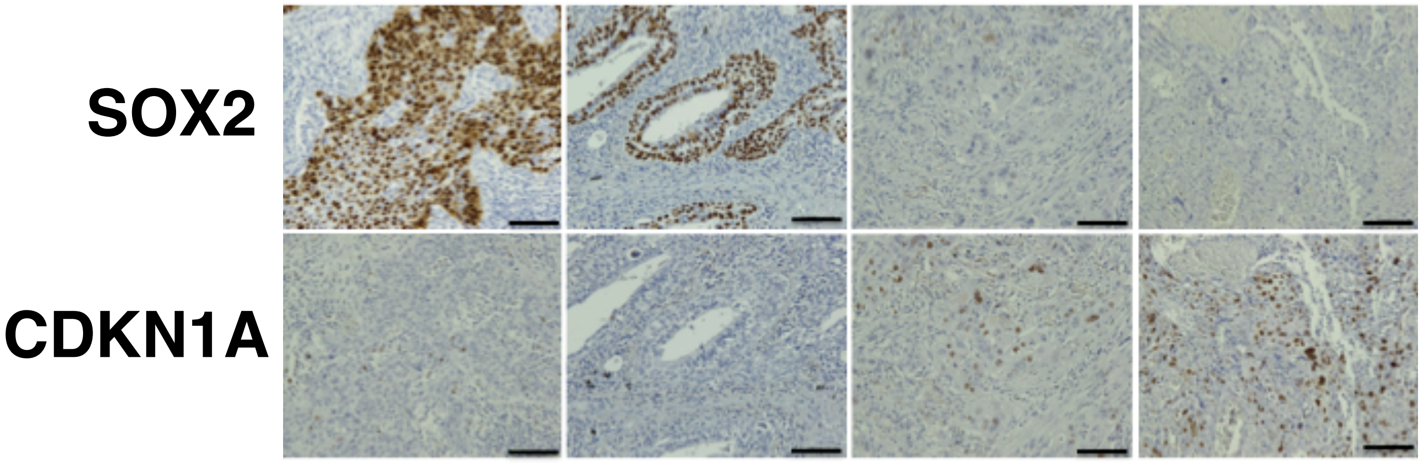

B

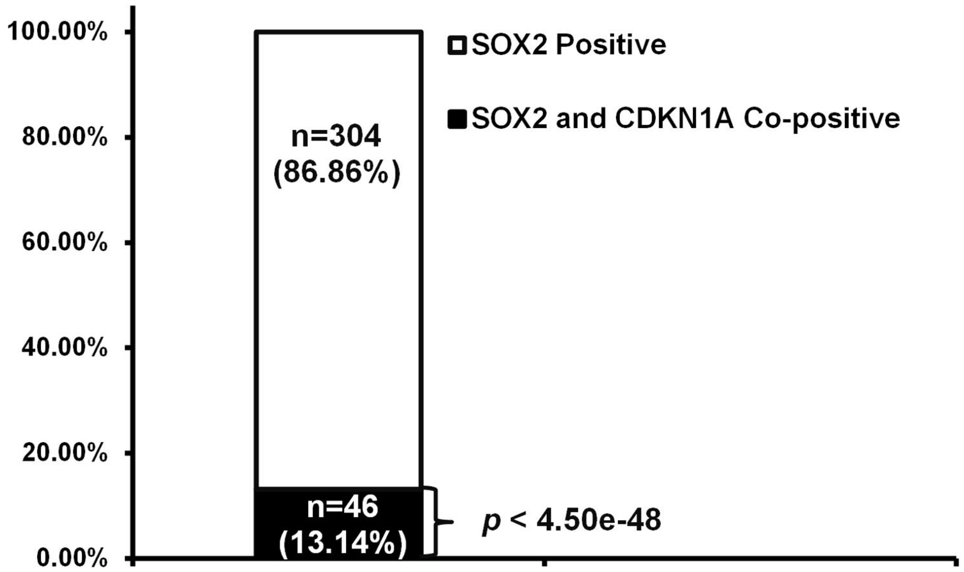

Supplementary Figure 1

Inversely related expression of SOX2 and CDKN1A in primary lung SCC A. Expression of SOX2 and CDKN1A was detected on serial sections of primary lung SCC by immunohistochemistry. Bar: 200 µm. **B.** 10 magnified views showing the expression of SOX2 as seen in A were randomly selected from lung SCC sections of each of 35 patients (out of 40). Lung SCC sections from 5 patients (out of 40) did not show the expression of SOX2. The number of CDKN1A (out of the 10 views) was counted for each serial section per patient that expressed SOX2. Among 350 SOX2 positive views, only 46 views (13.14%) were CDKN1A positive (co-positive for SOX2 and CDKN1A). One-tailed binomial test showed that the probability of co-positive was significantly less than 0.5 ($p<4.50e-48$), suggesting that expression of SOX2 and CDKN1A is inversely related in lung SCC.

Supplemental Figure 2

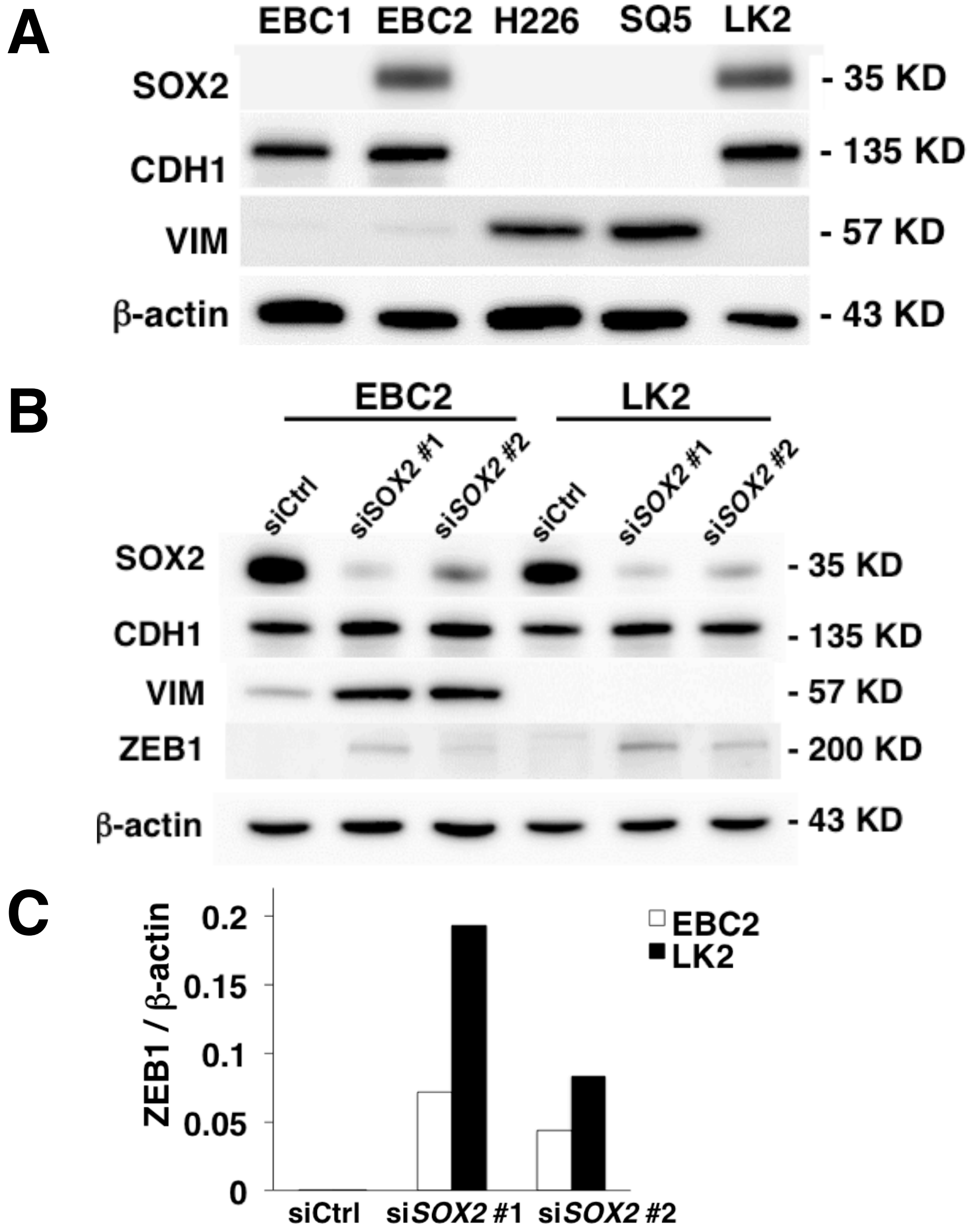

Supplemental Figure 2

SOX2 suppresses mesenchymal markers VIM and ZEB1 in EBC2 cells A. Expression of CDH1 (also known as E-cadherin; an epithelial marker) and VIM (also known as Vimentin; a mesenchymal marker) expression in lung SCC cells. CDH1 expression was detected by immunoblot analysis in EBC1, EBC2 and LK2 but not in H226 and SQ5 lung SCC cells. On the contrary, VIM expression was detected in CDH1-negative H226 and SQ5 cells but not in CDH1-positve in EBC1, EBC2 and LK2 lung SCC cells, indicating that the expression of CDH1 and VIM is mutually exclusive in lung SCC cells. **B.** ZEB1 was induced by *SOX2* siRNAs (si*SOX2* #1 and *siSOX2* #2) 48 hours after transfection in both EBC2 and LK2 lung SCC cells; however VIM was induced by the *SOX2* siRNAs only in EBC2 cells. Non-targeting siRNA was used as a control (siCtrl). **C.** Expression of ZEB1 was normalized by β-actin for each sample after the quantification using densitometric scanning with Adobe Photoshop CS5 Extended edition (Adobe Systems Inc., San Jose, CA, USA). Shown is the normalized expression of ZEB1 in EBC2 and LK2 lung SCC cells. ZEB1 was induced by the *SOX2* siRNAs in both EBC2 and LK2 cells.

Supplemental Figure 3

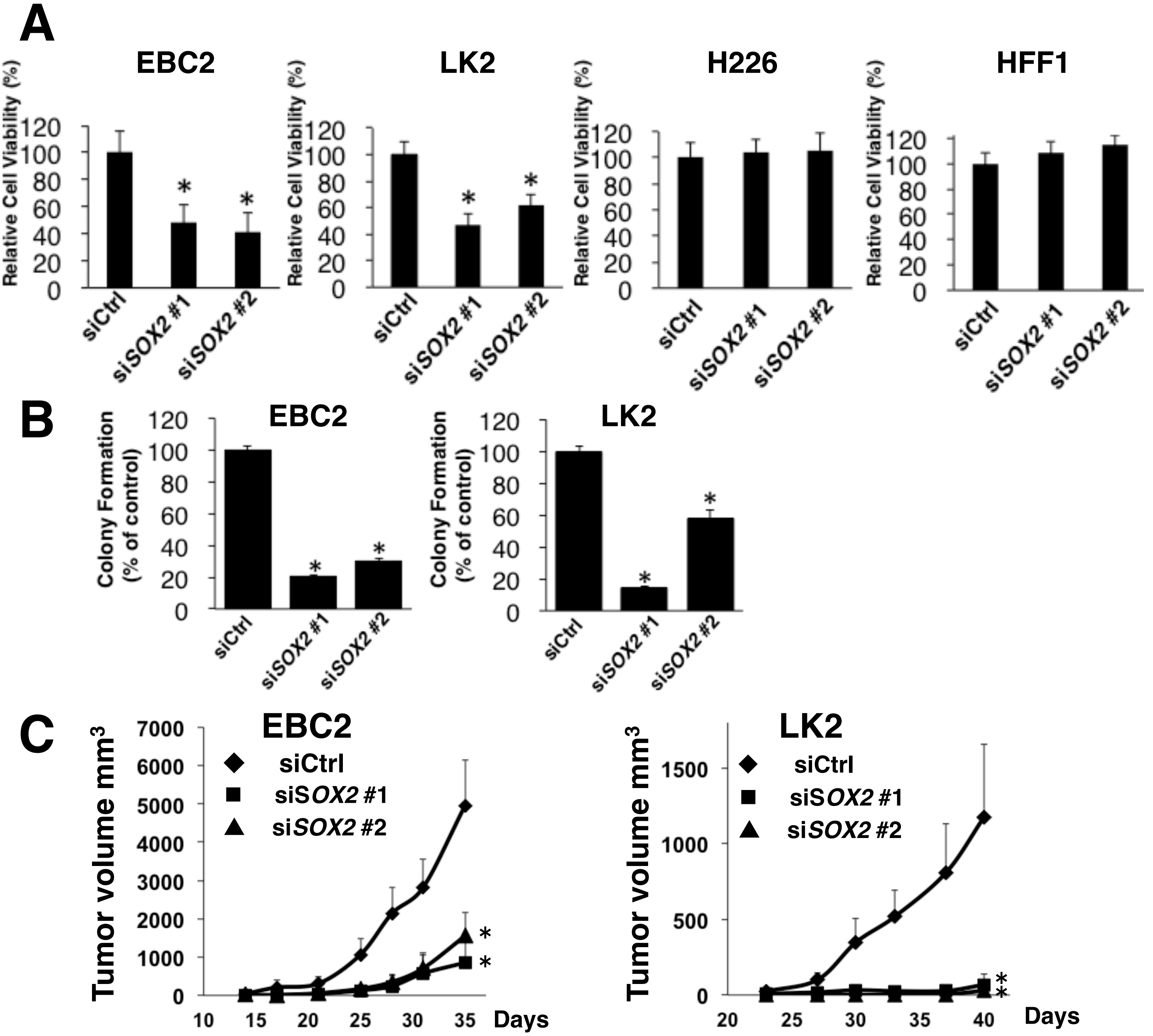

Supplemental Figure 3

SOX2 is required for cell viability, colony formation and tumor growth of lung SCC cells *in vitro* and *in vivo* **A.** *SOX2* siRNAs (si*SOX2* #1 and si*SOX2* #2) inhibited the cell growth of SOX2-expressing lung EBC2 and LK2 lung SCC cells. In contrast, *SOX2* siRNAs did not affect cell viability in SOX2-negative H226 lung SCC cells and in normal human foreskin fibroblast (HFF1) cells. Non-targeting siRNA was used as a control (siCtrl). Cell viability was assessed using a TC20 automated cell counter 48 hours after siRNA transfection. Statistical significance was defined as $p < 0.01$ (*). **B.** Colony formation of LK2 and EBC2 lung SCC cells treated with 100 pmol of *SOX2* siRNAs. Non-targeting siRNA was used as control (siCtrl). 14 days after treatments, cells were fixed and stained with Diff-Quik. Mean colony number was derived from quantitation of triplicate dishes for each treatment and was arbitrarily set to 100%. Statistical significance was defined as $p < 0.01$ (*). **C.** Volume of the tumors derived from EBC2 and LK2 lung SCC cells transfected with *SOX2* siRNAs is shown. The volume was monitored over time (days) after inoculation of tumor cells. Six mice were studied in each group. Tumor growth is expressed as mean tumor volume; bars represent SD. Statistical significance was defined as $p < 0.01$ (*).

Supplemental Figure 4

EBC2

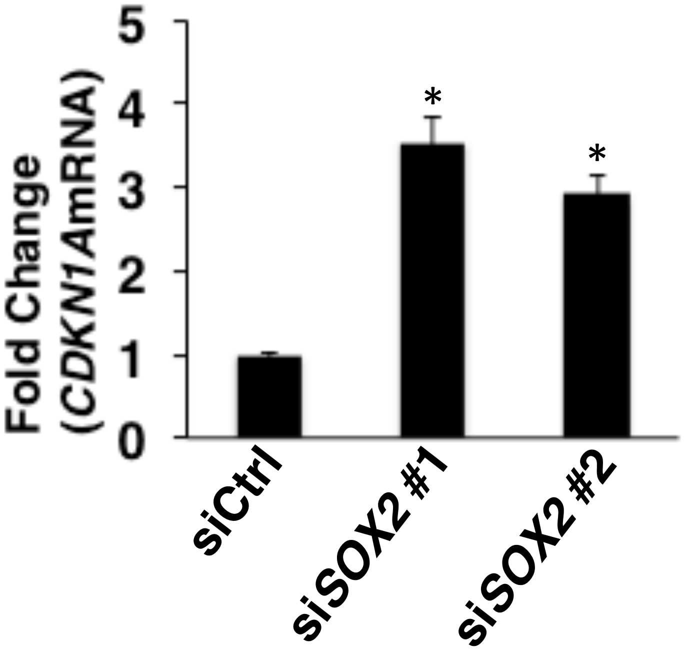

Supplemental Figure 4
Silencing SOX2 induced *CDKN1A* mRNA in EBC2 lung SCC cells in the presence of cycloheximide EBC2 cells were treated with 10 µM of cycloheximide for 15 minutes, then 100 pmol of *SOX2* siRNAs were transfected. Non-targeting siRNA was used as control (siCtrl). Total RNAs were extracted 48 hours after transfection. Real time PCR was performed as described in Materials and Methods.

Supplemental Figure 5

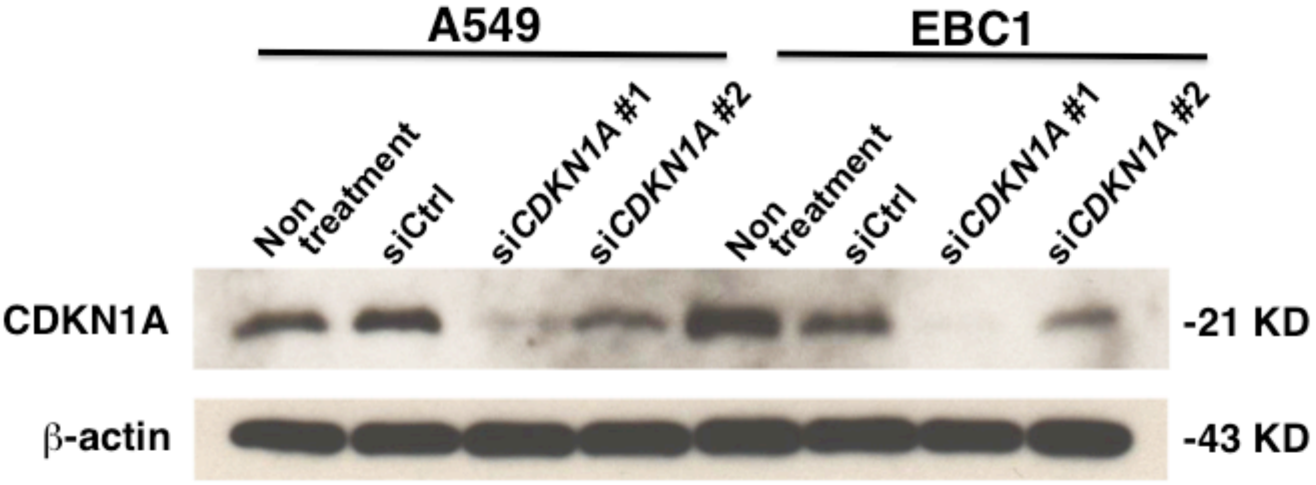

Supplemental Figure 5

Validation of siRNAs targeting CDKN1A CDKN1A was suppressed by two different *CDKN1A* siRNAs (si*CDKN1A* #1 and si*CDKN1A* #2) in A549 lung carcinoma cells and EBC1 lung SCC cells. Cells were harvested at 48 hours after 100 pmol of siRNA transfection. Protein expression was confirmed by immunoblot as described in Materials and Methods.

Supplemental Figure 6

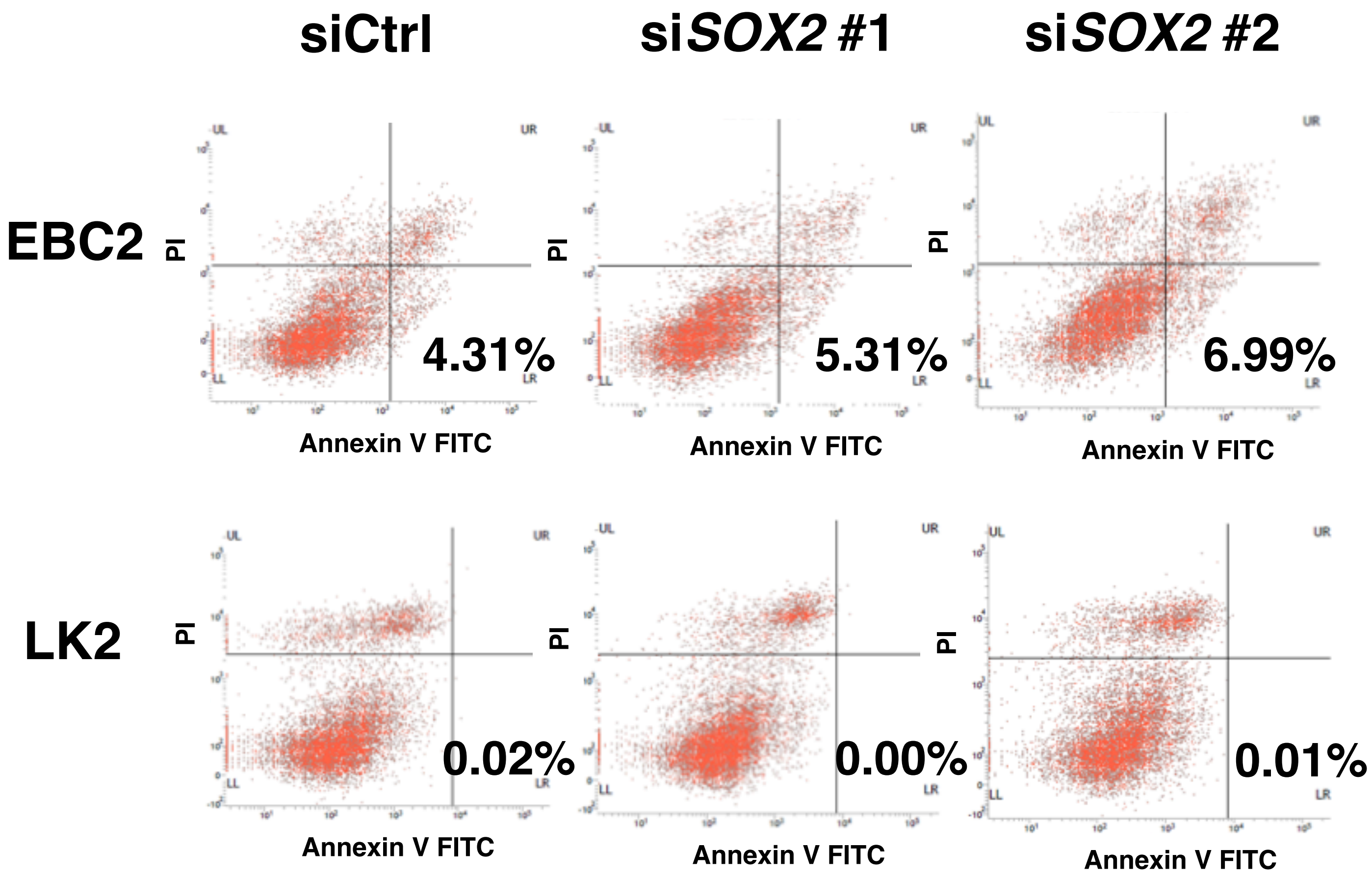

Supplementary Figure 6

SOX2 does not influence apoptosis in lung SCC cells Representative flow cytometric analysis of apoptotic cells 48 hours after *SOX2* siRNA (si*SOX2* #1 and si*SOX2* #2) transfection in LK2 and EBC2 lung SCC cells. Non-targeting siRNA was used as a control (siCtrl). Silencing SOX2 induced little apoptosis (Annexin V positive/PI negative population) in both LK2 and EBC2 lung SCC cells.
